# Value of serial echocardiography in diagnosing Kawasaki’s disease

**DOI:** 10.1007/s00431-020-03752-y

**Published:** 2020-09-21

**Authors:** Maria Hörl, Holger Michel, Stephan Döring, Markus-Johann Dechant, Florian Zeman, Michael Melter, Stephan Gerling

**Affiliations:** 1 University Children’s Hospital Regensburg (KUNO), University of Regensburg, Campus St. Hedwig, Regensburg, Germany; 2 Center for Clinical Studies, University Medical Center Regensburg, Regensburg, Germany

**Keywords:** Kawasaki disease, Aneurysm, Vasculitis, Coronary arteries, Immunoglobins intravenous, Echocardiography

## Abstract

Kawasaki disease (KD) is an acute vasculitis predominantly affecting the small arteries of young children. Up to 25% of untreated patients suffer from coronary artery (CA) complications. Early diagnosis and treatment is mandatory in incomplete KD to reduce the risk of coronary involvement. Between 2002 and 2018, 124 patients have been diagnosed suffering from KD at the University Children’s Hospital Regensburg (KUNO). We assessed luminal diameters of both CAs normalized as Z-scores by 2D-echocardiography. A total of 94 patients were analyzed. Of them, 31 (33%) were affected by an incomplete form of KD. In 24 children (26%), serial echocardiography was necessary in order to confirm diagnosis. Mean Z-scores for the left main coronary artery (LMCA), right main coronary artery (RMCA), and left anterior descending artery increased significantly between the initial (LMCA 0.79z, RMCA 0.15z, LAD 0.49z) and second (LMCA 1.69z, RMCA 0.99z, LAD 1.69z) examination (*p* < 0.05).

*Conclusion*:To confirm diagnosis of KD, it might not be necessary to detect dilation or aneurysms. Our observation suggests that patients suspected having KD should be monitored with serial echocardiography in order to detect a possible enlargement of the CA diameters, even if Z-scores are within the normal range.

**Table:** 

**What is Known:** • *Kawasaki disease (KD) is an acute vasculitis predominantly affecting the small arteries of young children. Up to 25% of untreated patients suffer from coronary artery (CA) complications.* *• Due to less classic clinical criteria in patients with incomplete KD, the risk for CA pathology is even higher.*	
**What is New:** *• A significant progression of patients’ CA Z-scores in serial echocardiographic measurements may be helpful to ensure diagnosis of KD early even if Z-scores are within the normal range.* *• Twenty-seven patients (90%) with incomplete KD could be diagnosed within 10 days of fever, early enough to prevent significantly higher rates of CA aneurysm.*

**What is Known:**

• *Kawasaki disease (KD) is an acute vasculitis predominantly affecting the small arteries of young children. Up to 25% of untreated patients suffer from coronary artery (CA) complications.*

*• Due to less classic clinical criteria in patients with incomplete KD, the risk for CA pathology is even higher.*

**What is New:**

*• A significant progression of patients’ CA Z-scores in serial echocardiographic measurements may be helpful to ensure diagnosis of KD early even if Z-scores are within the normal range.*

*• Twenty-seven patients (90%) with incomplete KD could be diagnosed within 10 days of fever, early enough to prevent significantly higher rates of CA aneurysm.*

## Introduction

Kawasaki disease (KD) is an acute vasculitis predominantly affecting small- and medium-sized arteries of young children. In developed nations, it is the leading cause of pediatric acquired heart disease [1]. The vascular inflammation process affects the arterial vessels of all the body regions, but the most serious manifestation is the affection of coronary arteries [2]. During the acute phase, up to 25% of untreated or late treated patients suffer from complications such as coronary artery dilation and coronary artery aneurysms related with increased morbidity and mortality [3].

The etiology of KD is still unknown, and a causative agent could not be found yet [4]. The ethnical distribution pattern suggests a genetic predisposition, with a varying incidence of 108–239.6 in Asia compared to 4.5–11.4 per 100,000 in Europe and 7.2–9 per 100,000 children younger than 5 years in Germany [5–9].

Since 1967, when Dr. Tomisaku Kawasaki—a Japanese pediatrician—first described the disease, no specific laboratory or diagnostic test could be developed [10]. Due to the similarity of the clinical manifestation with other infectious diseases, a lot of differential diagnosis must be excluded [11]. Particularly in infants and children older than 5 years who do not show the classical symptoms of the disease, called incomplete KD, diagnosing KD with clinical findings alone is even more challenging.

Due to less classic criteria in these patients with incomplete KD, they are at a higher risk to suffer from cardiac complications [12–14]. Therefore, cardiovascular imaging modalities, such as transthoracic echocardiography (TTE), magnetic resonance imaging (MRI), computed tomographic angiography (CTA), and invasive angiography, may help to diagnose KD early and prevent coronary artery complications and related evolving consequences [15].

To diagnose coronary artery involvement by using echocardiography, diameters are normalized for BSA as Z-scores. The Z-score is a classification system that provides the standard deviation of a value compared to a normally distributed population and allows comparison and standardization of measurements. Z-scores are available for proximal segments of both main coronary arteries [16].

We hypothesized that monitoring Z-scores in serial TTE will be helpful to diagnose children with incomplete KD before they will develop severe involvement of the coronary arteries. To determine the role of TTE in diagnosing KD, we performed serial echocardiography when there was suspicion of KD in infants and children admitted to our hospital.

## Methods

### Study population

The retrospective analyzed data has been collected from clinical records of patients at the children’s university hospital Regensburg (KUNO-Clinic St. Hedwig). We included all 124 patients diagnosed with KD aged from 2 months to 17 years between March 2002 and May 2018. All KD diagnoses were retrospectively determined according to the current AHA guidelines [17]. Complete KD was classified if children suffered from fever for five or more days, were unresponsive to systemic antibiotic therapy, and showed at least four major symptoms. If patients showed fever at least 5 days but less than four major symptoms, they were defined as incomplete KD patients if compatible laboratory results or echocardiographic abnormalities such as dilation, ectasia, or aneurysms could be identified.

The study is in compliance with the Declaration of Helsinki and was approved by the institutional review board of the University of Regensburg (file number 14-101-0206). On admission to our hospital, we collected demographic data, and records on medical, personal, and family history were obtained. Laboratory tests like complete blood count; serum electrolytes; the liver enzymes aspartate aminotransferase (AST), alanine aminotransferase (ALT), and gammaglutamyl transpeptidase (γGT); and the inflammatory marker C-reactive protein (CRP) were performed as part of our clinical routine. To estimate the body surface area (BSA) based on the children’s height and weight, we applied the formula of Haycock et al. [18]. We determined the first reported day of fever as first day of illness. Our patients were classified according to the AHA guidelines [17]. Complete KD was diagnosed if children suffered from fever for five or more days, were unresponsive to systemic antibiotic therapy, and showed at least four major symptoms. If patients showed fever at least 5 days but less than four major symptoms, they were diagnosed having incomplete KD if compatible laboratory results or echocardiographic abnormalities such as dilation, ectasia, or aneurysms could be identified.

KD patients received intravenous immunoglobulins (IVIG) in a dose of 2 g/kg bodyweight as a single infusion over 10 h and 40–100 mg/kg bodyweight acetyl salicylic acid (ASA) in four divided doses within 24 h until they were afebrile for 48 h (to 72). We continued to apply ASA in a lower dose (3–5 mg/kg bodyweight once per day) for 6 to 8 weeks in order to make use of its antiplatelet effect. IVIG unresponsiveness was classified in patients who were suffering from recrudescent or persistent fever at least 36 h after finishing treatment. In this case, a second dose of IVIG or additional corticosteroids (prednisolone) in a dose of 2 mg/ kg bodyweight were applied. Additionally, we recorded the days from the onset of fever to the echocardiographic examinations and the date of the final diagnosis. Patients with a history of vascular or cardiovascular disease and hematological disorders were excluded from our study.

### Echocardiography

If there was a suspicion for KD, a pediatric cardiological evaluation including a 12-lead electrocardiogram and TTE was performed. Serial TTE was conducted if KD could not be diagnosed with clinical findings, laboratory tests, and basis echocardiography. Two-dimensional, M-mode, and Doppler echocardiography was performed (Xario XG, since 2013 Aplio 500 CV; Toshiba Medical Systems Corporation, Otawara, Japan) with a 6.5-, 5.0-, or 3.0-MHz transducer (PST65-BT, PST-50BT, PST-30BT; Toshiba). All subjects were examined in supine or left lateral decubitus position by experienced pediatric cardiologists (SG, HM, SD).

In accordance with the AHA scientific statement on KD [17], both proximal coronary arteries were examined in the parasternal short- or long-axis view. As they advise, measurements of the LMCA should be viewed with caution because of anatomic variations like a dominant left or right coronary artery system. The diameters of the right main coronary artery (RMCA), left main coronary artery (LMCA), the left anterior descending coronary artery (LAD), and the left circumflex coronary artery (LCX) were measured. In case of coronary artery aneurysms, the maximal diameter was used. The distance from the trailing edge of the near wall to the leading edge of the far wall was measured as the coronary artery diameter 5 mm distal the ostium [19]. If there was a short stem of the LMCA (< 5 mm), the diameter was measured in the middle between the ostia and the branching. As it is uncommon to develop involvement only in the distal segments of coronary arteries, we considered patients with normal proximal diameters having distal segments within the range [17]. For statistical interpretation, we used averaged values from two or three single measurements of the coronary artery diameter. To figure out coronary artery involvement for classification and long-term follow up, we assessed luminal dimensions normalized for BSA as Z-scores according to Dallaire et al. [16].

We determined a mean Z-score < 2.0 SD as normal, whereas a mean Z-score between 2.0 and < 2.5 SD was declared as dilation. Small aneurysm of coronary arteries was defined as a mean Z-score 2.5 to < 5.0, medium aneurysm 5.0 to < 10, and large aneurysm from ≥ 10 or absolute dimension ≥ 8 mm [17]. All studies were digitally stored for off-line analysis, retrospective re-evaluation, and follow-up examinations.

### Statistics

Demographic and epidemiological characteristics are presented using absolute and relative frequencies for categorical variables and median (1. quartile, 3. quartile) for continuous data. Comparisons between complete and incomplete KD were performed using a chi-squared test of independence or the Mann-Whitney *U* test, respectively. For the analysis of changes in Z-scores of coronary arteries, paired Student’s *t* tests were applied. Z-score categories were compared between complete and incomplete KD using the chi-square test of independence. A *p* value < 0.05 was considered statistically significant. Statistical analysis was performed using IBM SPSS Statistics 25 software.

## Results

**Fig. 1:**
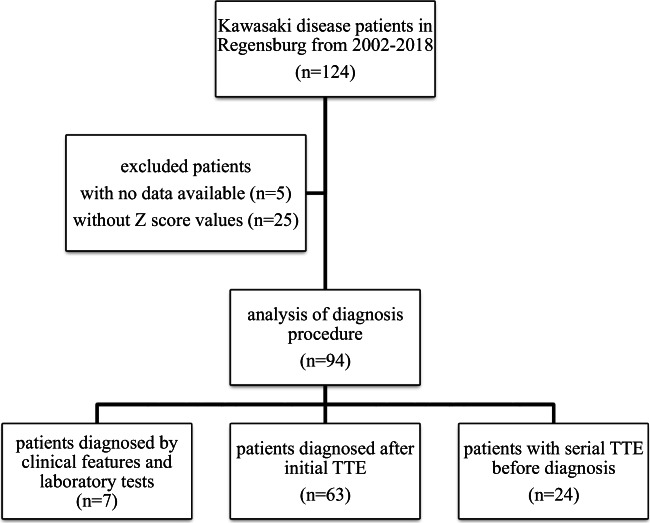
Flow chart of 124 Kawasaki disease patients. In 94 patients, Z-scores for coronary artery diameters were available. A serial echocardiography was performed in 24 children

During the study period, 124 patients were diagnosed suffering from KD. Five of them had to be excluded because of missing clinical reports or initial treatment before hospitalization in our clinic, and 25 patients had to be excluded because of missing echocardiographic results or values for calculating BSA, so we analyzed a total of 94 children (Fig. 1). The median age of all patients with acute phase of KD was 41 months, ranging from 2 months to 17 years. Thirty-one patients (33%) were older than 5 years, and 11 patients (12%) were less than 12 months old. As typically seen in patients with KD, 63 of the KD patients (67%) were male patients with a male-female ratio of 2:1. On average, patients were diagnosed having KD after a median of 6 days of illness (range 1–26 days). Eighty-one patients (86%) were diagnosed within the first 10 days of fever.

We compared clinical and epidemiological data of patients with complete and incomplete KD (Table 1). Complete KD showed in 63 of the 94 children (67%). The median interval between onset of fever and diagnosis was 6 days (range 2–26). Fifty-four patients (86%) out of all patients with complete KD were diagnosed early within the first 10 days of illness.

**Table 1:** Clinical and epidemiological data of 94 patients with Kawasaki disease

Variable	Complete KD (*n* = 63)	Incomplete KD (*n* = 31)	*p*
Male, *n* (%)	45 (71%)	18 (58%)	0.195
Age, months	40 (27; 67)	50 (13; 92)	0.958
BSA, m^2^	0.64 (0.55; 0.80)	0.63 (0.49; 0.89)	0.691
Maximum body temperature, °C	40 (39.7; 40.2)	40 (39.6; 40.5)	0.249
Duration of hospitalization, days	7 (6; 9)	7 (6; 10)	0.782
Duration of fever at diagnosis, days	6 (5; 8)	7 (6; 9)	0.141
Diagnosis ≤10 days of fever, n (%)	54 (86%)	27 (90%)	0.564
CRP, mg/dl	90 (52; 169)	100 (55; 193)	0.544
Hb, g/dl	10.4 (9.5; 11.2)	10.1 (9.0; 11.3)	0.250
WBC count, 10^9^/ L	14.1 (11.8; 19.6)	16.7 (10.7; 19.8)	0.763
Segmented neutrophils, %	70 (60; 76)	60 (53; 72)	0.024
Platelet count, 10^10^/L	56.9 (47.9; 76.9)	61.7 (33.4; 77.2)	0.627
Sodium, mmol/l	131 (128; 134)	131 (130; 134)	0.306
AST, U/l	43 (31; 75)	42 (34; 52)	0.565
ALT, U/l	49 (20;114)	21 (15; 42)	0.011
ɣGT, U/l	42 (17; 96)	22 (11; 83)	0.096

Clinical and epidemiological data were available for 94 KD patients. Data are expressed in median (q1; q3) maximum values of CRP, WBC, segmented neutrophils, platelets, AST, ALT, and ɣGT and minimum values of Hb and sodium of all laboratory results

*BSA* body surface area, *CRP* C-reactive protein, *Hb* hemoglobin, *WBC* white blood cell, *AST* aspartate aminotransferase, *ALT* alanine aminotransferase, *ɣGT* gammaglutamyl transferase

Of all 94 patients, 31 were affected by incomplete KD (33%). The median number of days with fever at diagnosis was 7 (range 1–26 days). So, the time of illness at diagnosis was not significantly longer compared to those with complete KD (*p* = 0.782). In the subgroup of patients with incomplete KD, 27 patients (90%) could be diagnosed within 10 days of fever. Comparing patients with complete and incomplete KD, 9 patients with complete KD (14%) and 3 patients with incomplete KD (10%) had delayed diagnosis after 10 days of fever (*p* = 0.564).

Laboratory results did not differ significantly between patients with complete and incomplete KD, except of a lower percentage of segmented neutrophils and ALT levels for patients with incomplete KD.

### Echocardiography

In our cohort, diagnosis could be established before a basis TTE was done in seven out of all KD patients (7.4%). These patients showed typical clinical features and laboratory test results. In 63 patients (67%), KD was diagnosed on the day of their initial echocardiography. The initial TTE was performed on the seventh day of fever (mean, range 1–26 days). Two patients were diagnosed on the 26th day of illness, because they presented late on the 21st and 25th days of fever respectively. Both showed coronary artery Z-scores between 2.5 and 5.0 SD but developed no segmental aneurysms.

We analyzed coronary artery Z-scores in Table 2. Of 63 patients with complete KD, 22 patients (35%) had no coronary artery involvement at all. Of 31 patients with incomplete KD, eight patients (25.8%) had no coronary artery involvement. Overall, coronary artery Z-scores were not significantly higher in patients with incomplete KD compared to patients with complete KD (*p* = 0.393).

**Table 2:** Coronary artery Z-score results of 94 patients with Kawasaki disease

Z-score	Complete KD (*n* = 63)	Incomplete KD (*n* = 31)	*p*
< 2.0 SD	22 (35%)	8 (25.8%)	0.393
2.0 to < 2.5 SD	14 (22%)	4 (12.9%)	
2.5 to < 5.0 SD	22 (35%)	16 (51.6%)	
≥ 5.0 SD	5 (8%)	3 (9.7%)	

Maximum Z-score results of 63 patients with complete and 31 patients with incomplete KD with available measurements for all coronary arteries. Statistical analysis by chi-square-test

**Fig. 2:**
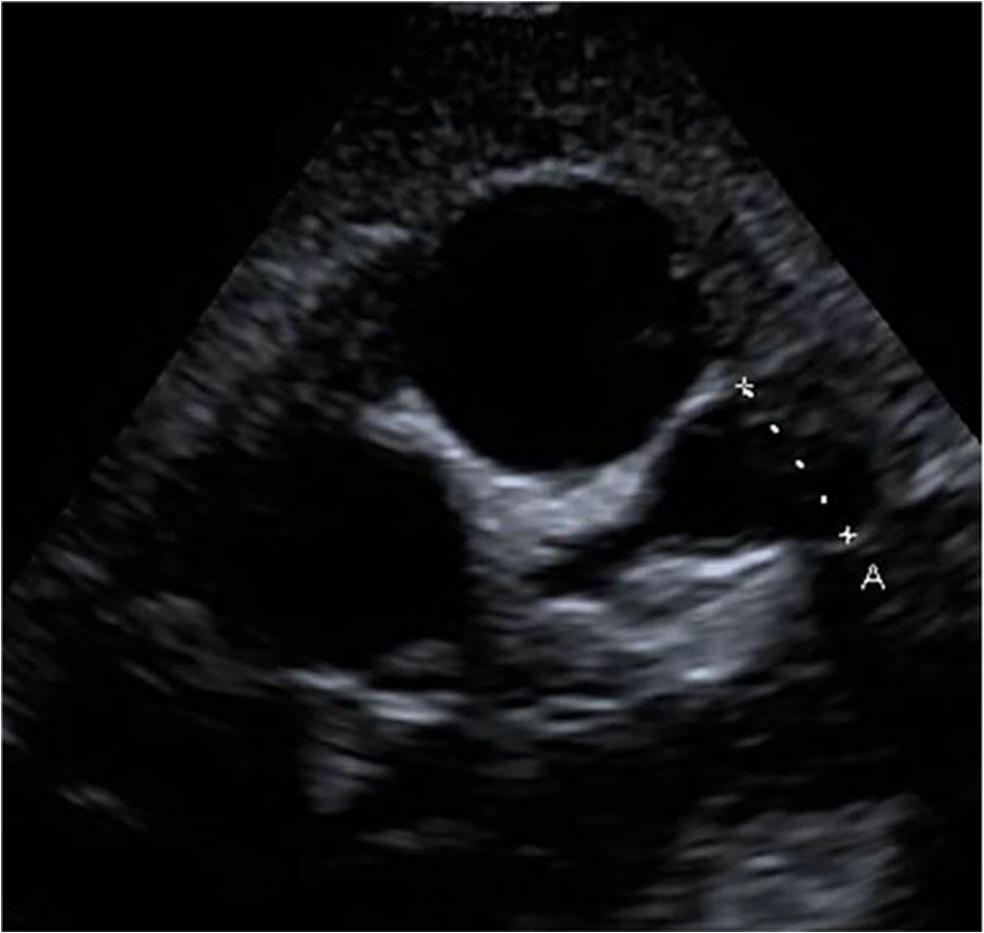
Transthoracic echocardiography, parasternal short-axis view. Giant aneurysm (A = 10 mm) of the LAD in a 3-year-old girl with Kawasaki disease

Of all patients, seven patients seemed to have a segmental dilation of the coronary arteries in echocardiography (Fig. 2). These findings were confirmed in a cardiac angiography during follow up.

### Serial echocardiography

We performed serial measurements of the coronary artery diameters if diagnosis of KD could not be established with clinical symptoms, laboratory results, and basis echocardiography. So, in 24 children (26%), serial echocardiography with two or more examinations was necessary to confirm diagnosis of KD. Out of this subgroup, 10 patients (42%) were affected by an incomplete form of KD, which is more than in the subgroup of patients without serial echocardiography (30% incomplete KD, *p* = 0.294). The remaining 14 patients (58%) in this subgroup with serial echocardiography before diagnosis were retrospectively classified having complete KD; at the time of basis echocardiography, diagnosis was not clear, and missing clinical symptoms occurred later on.

**Fig. 3:**
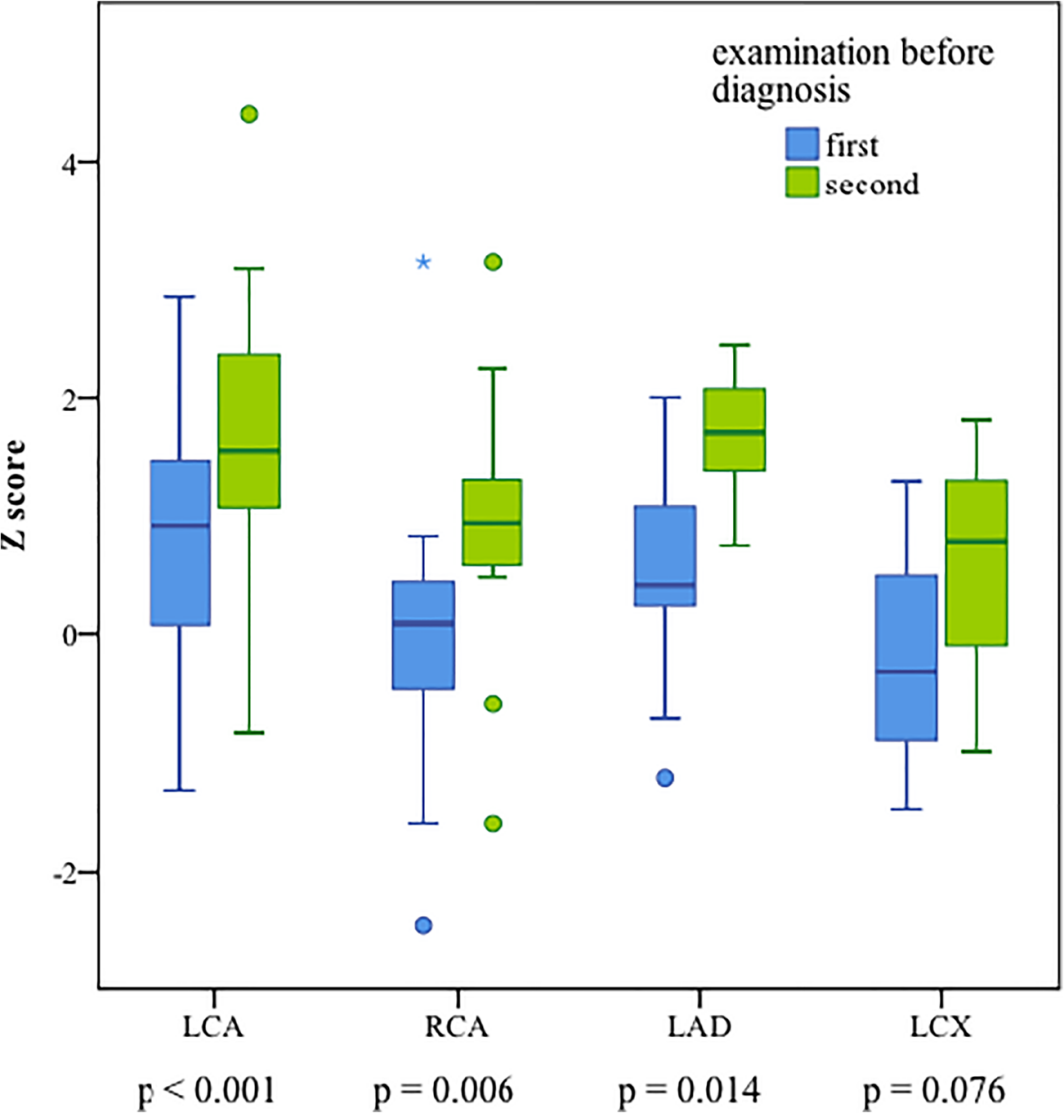
Coronary artery Z-score results for the left main coronary artery (LMCA, *n* = 21), right main coronary artery (RMCA, *n* = 17), left anterior descending artery (LAD, *n* = 9), and left circumflex artery (LCX, *n* = 3) for KD patients out of the subgroup of children with serial TTE. Mean values of coronary artery diameters of the initial and second echocardiography prior to diagnosis. Central lines indicate median and IQR, T-bars show 5th to 95th percentile

Data for coronary artery Z-scores were available in 21 (88%) out of 24 patients in the subgroup of children with serial TTE. Mean coronary artery Z-scores for the LMCA (*n* = 21) increased significantly between the initial and second examination (*p* < 0.001). See Table 3 and Fig. 3. In 18 out of these 21 patients, LMCA Z-scores showed an increase, and in three patients, diameters remained unchanged or decreased. Mean coronary artery Z-scores for the RMCA (*n* = 17) also increased significantly between the initial and second TTE (*p* = 0.008). Twelve patients developed an increase of the RMCA Z-score, whereas five patients did not show progression of the RMCA diameter. The LAD Z-scores were available for nine patients, eight of them showed an increase of the Z-score, and in one patient, the LAD diameter did not change (*p* = 0.014). For LCX, coronary artery Z-scores were available just for three patients, and all of these patients showed an increase in the Z-score, which was, however, not statistically significant (*p* = 0.076). In total, we found increasing coronary artery Z-scores in 20 patients out of 21 patients (95%). When enlargement of the coronary artery diameter was detected, diagnosis was confirmed, and therapy could be initiated.

**Table 3:** Coronary artery Z-score results of serial echocardiography in 21 Kawasaki disease patients

	Initial TTE	Second TTE	Difference (∆Z-score)	*p*
LMCA (*n* = 21)	0.79 ± 1.12	1.69 ± 1.17	0.90 (95%-CI 0.51, 1.29)	< 0.001
RMCA (*n* = 17)	0.15 ± 1.23	0.99 ± 1.10	0.84 (95%-CI 0.25, 1.43)	0.008
LAD (*n* = 9)	0.49 ± 1.04	1.69 ± 0.53	1.20 (95%-CI 0.32, 2.08)	0.014
LCX (*n* = 3)	− 0.16 ± 1.39	0.54 ± 1.42	0.70 (95%-CI − 0.18, 1.58)	0.076

Coronary artery Z-scores for both examinations were available in 21 (88%) out of 24 patients in the subgroup of children with serial TTE. Mean Z-scores, ±SD, and difference between Z-scores (95%-CI) with *p* value of coronary artery results from two cardiac examinations before diagnosis

*LMCA* left main coronary artery, *RMCA* right main coronary artery, *LAD* left anterior descending artery, *LCX* left circumflex coronary artery

Although almost every patient out of this subgroup showed progression of coronary artery diameters, the average Z-scores before diagnosis were mainly within the normal range, and nine of them (38%) had no pathological coronary artery involvement at all. Seven patients (29%) had Z-scores between 2.0 and < 2.5 SD, and seven patients (29%) developed coronary artery Z-scores between 2.5 and < 5.0 SD. One patient (4%) had a coronary artery Z-score above 5.0 SD. The lower incidence of severe coronary artery involvement in this subgroup shows that the patient’s coronary artery diameters should be monitored, even if Z-scores are within the normal range (< 2.0 SD), to figure out if coronary artery involvement is in progress.

Most of these patients (*n* = 20, 83%) underwent echocardiography two times. In three patients (13%), three TTEs were carried out, and in one patient (4%), four TTEs were necessary to diagnose KD. In Table 3 and Fig. 3, we used the first two Z-score results that were documented. Mean interval between the initial and the second TTE was 1.8 days, with a minimum of 1 and a maximum of 4 days. It is noticeable that patients with greater intervals between the TTEs (three or more days) showed a greater increase of coronary artery Z-scores, but a significant increase in coronary artery diameters could already be identified after 24 h.

## Discussion

As Kawasaki disease has a relatively low incidence in Germany compared with other European countries and Asia [5–9], identifying a child suffering from KD remains difficult. No pathognomonic laboratory test to validate diagnosis of KD is available until now. Late diagnosis of KD after the 10th day of fever and delayed treatment are reported risk factors in developing coronary artery aneurysms [20–22]. In a long-term follow-up analysis (mean 10.2 years, range 1–36 years), Maccora et al. were able to demonstrate the benign course of KD even in coronary artery lesion patients, if timely treated [23]. This makes early diagnosis and treatment pivotal in order to prevent cardiac complications.

Additionally, in patients with an incomplete KD, it is even more challenging to diagnose KD due to less classic clinical criteria [24]. In our study cohort, one-third of the patients were affected by an incomplete form of KD, which is well comparable to former studies [25, 26].

Coronary artery involvement is one of the most severe complications for patients with KD. Capannari et al. proved high sensitivity and specificity of transthoracic echocardiography (TTE) in order to detect aneurysms in the proximal regions of the coronary arteries [27].

Recently, Fuse et al. demonstrated that changes in coronary artery Z-scores may be observed in the early phase of KD already. Coronary artery dilation occurred within the first 10 days of illness in 70% of the KD patients [28].

However, it is not well defined how serial TTEs can help in order to diagnose KD early, especially for those with incomplete KD. Out of the 31 patients with incomplete KD in our cohort, two patients (7%) were diagnosed by clinical features, and 19 (61%) were diagnosed performing echocardiography once. Using serial echocardiography, diagnosis was confirmed in another ten patients (32%) with incomplete KD. So, in incomplete forms of KD, an echocardiography was diagnostic in 93% of our patients. By applying serial TTE, we achieved that the proportion of patients who were diagnosed after the 10th day of illness was 14% in the group with complete KD and just 10% in the group with incomplete KD respectively. Additionally, the median number of days with fever at diagnosis in patients with incomplete KD was not significantly longer than in patients with complete KD. Consequently, patients with incomplete KD did not suffer from a significant delay in diagnosis in our cohort.

The number of patients with coronary artery Z-scores between 2.0 SD and 2.5 SD was lower in incomplete KD compared to patients with complete KD, whereas the incidence of coronary artery Z-scores above 2.5 SD was higher in incomplete KD.

In our cohort, of all patients with Z-scores above 2.5 in at least one coronary artery, seven patients appeared to have a segmental dilation, and all of them could be confirmed as aneurysms in cardiac catheter examination. Only one patient with incomplete KD suffered from a medium-sized aneurysm.

The AHA reported [24] that coronary artery aneurysms normally do not occur within the first week of fever in KD. In the initial examination, roughly 25% of KD patients younger than 6 months showed normal echocardiography results but presented abnormalities in echocardiography mostly in the following 2 weeks [29].

In a recent prospective study from China, Liu et al. stated that patients with KD and greater initial aneurysm size, CA aneurysm progression, more involved coronary arteries, and lower lymphocyte proportion may require intensive cardiac monitoring and adjuvant therapies. Improved echocardiographic techniques and routine use of CA Z-scores for definition of CA aneurysm may lead to identification of small CA aneurysm, and this may also contribute to the higher CA aneurysm regression [30].

We could detect early coronary artery involvement by demonstrating a significant increase of coronary artery diameters. We propose that a significant progression in the patients’ coronary artery Z-scores may be helpful to ensure diagnosis of KD early, in incomplete cases, where classic symptoms are missing, as well as in patients with complete KD, when some classic symptoms occurred later on. To confirm diagnosis of KD, it might not be necessary to detect dilation or aneurysms. Our observation suggests that patients suspected having KD should be monitored with serial echocardiography in order to detect a possible enlargement of the coronary artery diameters, even if Z-scores are within the normal range.

However, it should be mentioned that a single TTE without pathological findings cannot exclude KD, whereas abnormalities identified in the echocardiography ensure the diagnosis [31]. It is mandatory that in cases with clinical or laboratory findings strongly suggesting KD, therapy should be started even with normal echocardiographic results.

Our study must be viewed considering some limitations. First, this is a retrospective study, which might have introduced bias in the results. Second, the study included a relatively small number of patients with Kawasaki disease due to the low prevalence of the disease in Germany [9] and took place at a single center. Additionally, due to the prolonged observation time, at the beginning of the study period, coronary artery diameters—especially of the LAD and LCX—were, unfortunately, not well documented. Thus, we had to exclude patients due to missing echocardiography data as well as missing documentation of body measurements, as both might have affected the results.

## Conclusion

The results of our study support the current American Heart Association recommendations [17] to use TTE as an important non-invasive diagnostic tool for Kawasaki disease. In the absence of pathognomonic tests, we were able to diagnose Kawasaki disease by a significant progression in coronary artery diameter Z-scores in serial TTE. A substantial number of patients suffering from incomplete Kawasaki disease were diagnosed based on echocardiographic criteria, early enough to prevent significantly higher rates of coronary artery involvement. Further studies with larger cohorts of Kawasaki disease patients are needed to confirm our findings.

## Abbreviations

BSA: Body surface area
CA: Coronary artery
Hb: Hemoglobin
KD: Kawasaki disease
LMCA: Left main coronary artery
LAD: Left anterior descending artery
LCX: Left circumflex coronary artery
RMCA: Right main coronary artery
TTE: Two-dimensional transthoracic echocardiography
WBC: White blood cell
